# Pentoxifylline for Anemia in Chronic Kidney Disease: A Systematic Review and Meta-Analysis

**DOI:** 10.1371/journal.pone.0134104

**Published:** 2015-08-03

**Authors:** Davide Bolignano, Graziella D’Arrigo, Anna Pisano, Giuseppe Coppolino

**Affiliations:** 1 CNR- Institute of Clinical Physiology, Reggio Calabria, Italy; 2 Nephrology and Dialysis Unit, University Hospital, Catanzaro, Italy; University of Sao Paulo Medical School, BRAZIL

## Abstract

**Background:**

Pentoxifylline (PTX) is a promising therapeutic approach for reducing inflammation and improving anemia associated to various systemic disorders. However, whether this agent may be helpful for anemia management also in CKD patients is still object of debate.

**Study Design:**

Systematic review and meta-analysis.

**Population:**

Adults with CKD (any KDOQI stage, including ESKD patients on regular dialysis) and anemia (Hb<13 g/dL in men or < 12 g/dL in women).

**Search Strategy and Sources:**

Cochrane CENTRAL, EMBASE, Ovid-MEDLINE and PubMed were searched for studies providing data on the effects of PTX on anemia parameters in CKD patients without design or follow-up restriction.

**Intervention:**

PTX derivatives at any dose regimen.

**Outcomes:**

Hemoglobin, hematocrit, ESAs dosage and resistance (ERI), iron indexes (ferritin, serum iron, TIBC, transferrin and serum hepcidin) and adverse events.

**Results:**

We retrieved 11 studies (377 patients) including seven randomized controlled trials (all comparing PTX to placebo or standard therapy) one retrospective case-control study and three prospective uncontrolled studies. Overall, PTX increased hemoglobin in three uncontrolled studies but such improvement was not confirmed in a meta-analysis of seven studies (299 patients) (MD 0.12 g/dL, 95% CI -0.22 to 0.47). Similarly, there were no conclusive effects of PTX on hematocrit, ESAs dose, ferritin and TSAT in pooled analyses. Data on serum iron, ERI, TIBC and hepcidin were based on single studies. No evidence of increased rate of adverse events was also noticed.

**Limitations:**

Small sample size and limited number of studies. High heterogeneity among studies with respect to CKD and anemia severity, duration of intervention and responsiveness/current therapy with iron or ESAs.

**Conclusions:**

There is currently no conclusive evidence supporting the utility of pentoxifylline for improving anemia control in CKD patients. Future trials designed on hard, patient-centered outcomes with larger sample size and longer follow-up are advocated.

## Introduction

Anemia is exceedingly frequent among subjects with chronic kidney disease (CKD). The prevalence of this condition increases as renal function decreases and more than one half of patients with end stage kidney disease (ESKD) are estimated to have an anemic state [1].

Although the advent of erythropoiesis stimulating agents (ESAs) represented an authentic revolution for treating anemia in CKD, the rising incidence of progressive CKD has led to an overwhelming demand for such agents with overall costs that are expected to increase dramatically in the next future [2, 3]. Furthermore, higher ESAs doses have been associated with an increased risk of death and adverse cardiovascular outcomes [4, 5].The search of alternative therapies for optimizing anemia management is thus advocated, particularly in subjects with co-morbid conditions predisposing to poor responsiveness to ESAs or iron supplements. Pentoxifylline, a non-specific phosphodiesterase inhibitor endowed with several hemorrehologic properties, has been shown to improve anemia associated to various conditions such as massive hemolysis [6], chronic hepatitis [7], myelodysplastic syndrome [8] and sickle cells disease [9].

There is now sparse evidence indicating that pentoxifylline might improve anemia parameters also in CKD patients by ameliorating inflammation [10]. However, promising results obtained in small prospective uncontrolled studies were not fully confirmed by more recent randomized trials. As a result, the effective utility of this agent for anemia management in CKD is still object of debate.

We therefore aimed at performing a systematic review and meta-analysis for clarifying benefits and harms of pentoxifylline supplements for anemia control in CKD.

### Methods

This systematic review and meta-analysis was conducted according to a previously published protocol (http://www.crd.york.ac.uk/PROSPERO/display_record.asp?ID=CRD42015019042)

### Data source and search strategy

Ovid-MEDLINE, EMBASE, PubMed and CENTRAL databases were searched for English-language articles without time restriction up to June 26, 2015 through focused, high sensitive search strategies (S1 Table). References from relevant studies were screened for supplementary articles. The search was designed and performed by one Author (DB).

### Study selection and data extraction

We included any randomized controlled trial (RCT), quasi-RCT (trials in which allocation to treatment was made by alternation, use of alternate medical records, date of birth or other expected methods), prospective or retrospective study providing information on the effects of pentoxifylline on anemia parameters in patients with CKD. Pentoxifylline treatment was considered regardless of dosage or duration of administration. For controlled studies any possible comparator, including placebo or no therapy, was considered. Data on routine treatment, including but not limited to iron supplements and erythropoiesis stimulating agents (ESAs) were recorded for possible exploratory analyses. Studies were considered without follow-up duration restrictions.

The presence of CKD was defined according to the National Kidney Foundation-Kidney Disease Outcomes Quality Initiative (NKF KDOQI) guidelines [11] by a reduced glomerular filtration rate (GFR)<90 mL/min/1.73 m^2^ and/or by the persistence of urinary abnormalities such as albuminuria, proteinuria or hematuria for at least 3 months. Studies focusing on end-stage kidney disease (ESKD) patients needing chronic renal replacement therapy by hemo- or peritoneal-dialysis were also included.

Anemia was defined as the presence of hemoglobin <13 g/dL in men or < 12 g/dL in women.

Studies were excluded if: 1) pentoxifylline was tested for improving anemia associated to other diseases (e.g. hematological, liver or vascular disorders); 2) dealing with CKD patients on acute renal replacement therapy (e.g. acute dialysis or peritoneal dialysis); 3) not providing long term data on the outcomes of interest (see below). Studies where at least part of the population fulfilled the above criteria were included in the review.

The primary endpoint of interest was end of treatment hemoglobin (Hb). Secondary outcomes were hematocrit (Hct), ESA requirement (dosage) and resistance (ERI) and iron indexes such as serum ferritin, total serum iron, total iron binding capacity (TIBC), transferrin (either as absolute levels or % of saturated transferrin, TSAT) and serum hepcidin. Clinically relevant adverse events were also recorded. Titles and abstracts were screened independently by two authors (GD and AP) who discarded studies that were not pertinent to the topic. Case reports, reviews, editorials, letters and studies performed on children (age<18) were excluded from qualitative analyses but screened for potential additional references. Two Authors (GD and AP) independently assessed the retrieved abstracts and the full text of these studies to determine eligibility according to the inclusion/exclusion criteria.

A third reviewer (DB) solved possible discrepancies on study judgments. Data extraction and analysis were performed by two reviewers (GD and AP) and independently verified by another (DB).

### Data analysis

Cumulative meta-analyses were performed if data on the same outcome were provided by more than one study. In order to maximize information, data on outcomes reported by single studies or in a descriptive way were reported narratively. The mean difference (MD) was used to assess the effects of treatment on continuous variables with the same scale. For variables reported with different scales (e.g. ESAs dosage) the standardized mean difference (SMD) was used. The relative risk (RR) was calculated for dichotomous outcomes (e.g. adverse events). Data were pooled using the random-effects model. To ensure robustness of the model and susceptibility to outliers pooled data were also analyzed with the fixed-effect model. Data available as median and range were converted to mean and SD by applying the Hozo formula [12].

Heterogeneity was assessed by the Chi^2^ test on N-1 degrees of freedom, with an alpha of 0.05 considered for statistical significance and the Cochrane-I^2^ [13]. I^2^ values of 25%, 50% and 75% were considered to correspond to low, medium and high levels of heterogeneity, respectively.

Possible sources of heterogeneity were explored by sensitivity and, if possible, by subgroup analyses and could be related to study design, duration of follow-up, administered dose of pentoxifylline, population characteristics (e.g. severity of CKD, dialysis vs. conservative treatment), baseline Hb levels and iron status, concomitant treatment with ESAs or iron supplements.

Statistical analyses were performed using Review Manager (RevMan; Version 5.3. Copenhagen: The Nordic Cochrane Centre, The Cochrane Collaboration, 2014) and Comprehensive Meta-analysis (Version 2.2, 2005; Biostat, Englewood, NJ).

### Quality and risk of bias assessment

The quality of RCTs was assessed by using the checklist developed by the Cochrane Renal Group which evaluates the presence of potential selection bias (random sequence generation and allocation concealment), performance bias (blinding of investigators and participants), detection bias (blinding of outcome assessors), attrition bias (incomplete outcome data), reporting bias (selective reporting) and possible other sources of bias. In addition, possible selection bias or confounding by indication was evaluated for non-randomized studies.

## Results

### Search results

**Fig 1:**
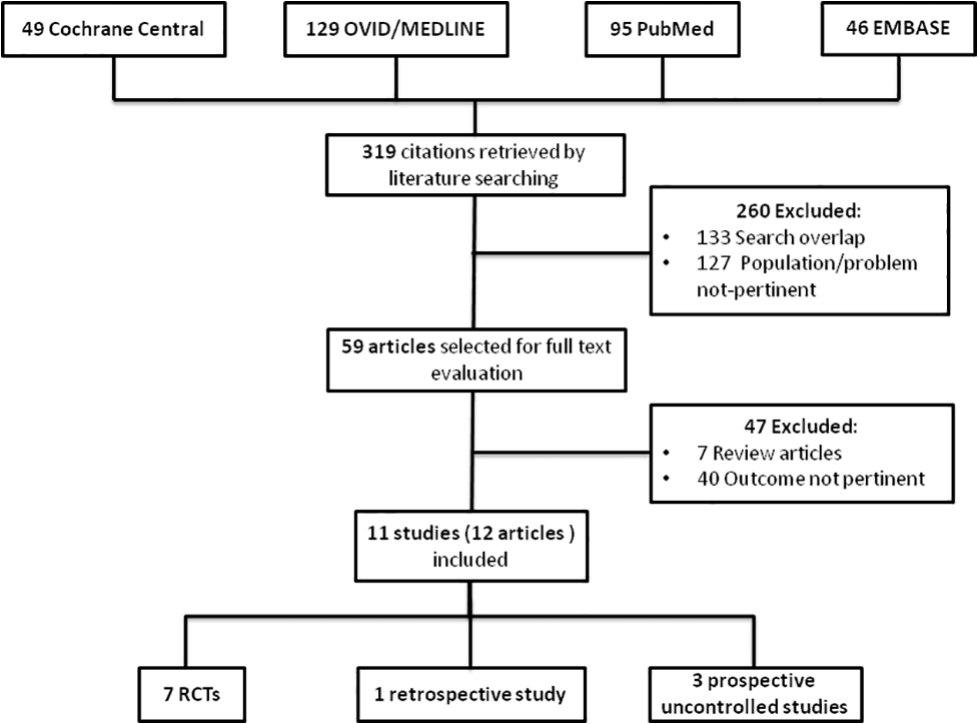
Study selection flow.

The flow diagram of the selection process is depicted in Fig 1. Three hundred and nineteen potentially relevant references were initially retrieved. By screening titles and abstracts, a total of 260 citations were excluded because of search overlap or because dealing with the wrong population/intervention. Amongst the 59 studies selected for full text examination, 47 studies were excluded because: not reporting outcomes pertinent to the topic (n = 40) or because reviews with no original data to be extracted (n = 7). A total of 12 articles referring to 11 full studies were reviewed in detail and included in the review.

Main characteristics of these studies are summarized in Tables 1 and 2.

**Table 1:** Summary of the main characteristics of the non-randomized studies reviewed.

Study, year (ref)	Design	Study population	Population characteristics	Duration	Intervention	Outcome(s)	Results
Cooper et al. 2004 [14]	Prospective uncontrolled study	ESKD patients with poor response to ESA (both on HD or conservative treatment)	N = 12,Men(%) = 50, Age = 54±10 yrs, ESA dose (IU/kg/wk) = 294±125-Ferritin (mcg/L) = 472±28	4 months	Pentoxifylline400 mg/day	Hemoglobin (g/dL)	Significant increase from 9.5 ±0.9 to 11.7±1.0 g/dL (p = 0.0001)
Ferrari et al. 2010 [15]	Prospective uncontrolled study	Patients with CKD stage 4–5 not on dialysis with iron deficiency	N = 10, Age = 64±7 yrs, Hb (g/L) = 111 ± 5, Ferritin (mg/L) = 92 ±26, TSAT (%) 13 ± 3	4 weeks	Pentoxifylline400 mg/day	Hemoglobin (g/L)	Significant increase from 111 ±5 to 123±6 (p<0.01)
						TSAT (%)	Significant increase from 13± 3 to 20±5 (p<0.003)
						Ferritin (mg/L)	No changes from baseline values
Mora-Gutiérrez et al. 2013 [16]	Retrospective case-control study	HD patients aged ≥17 yrs with dialysis vintage> 3 months	N = 36, Men(%) = 56, Age = 59.4, DM (%) = 14	6–32 months	Pentoxifylline 400 mg/twice day (n = 18)-Standard therapy (Controls; n = 18)	Hemoglobin (g/dL)	-Significant increase in PTX group (from 9.3±1 to 11.2±1, p<0.001)-No changes in control group-No difference between groups at the end of the study (11.2±1 vs 11.5±0.7 in PTX vs controls, p = 0.415)
						ESAs dose (mcg/month)	-Significant decrease in PTX group (from 170.8±66.6 to 106.1±89.7, p<0.002)-No changes in control group
						Ferritin (ng/mL)	Significant difference between groups at the end of the study (648.3 ±221.6 vs 320.2 ±168.6 in PTX vs. controls, p = 0.008)
Mohammadpour et al. 2014 [17]	Prospective uncontrolled study	Patients on HD for at least 6 months with anemia (Hb <10.7 g/dL) unresponsive to ESA at a dose of ≥12.000 IU/week	N = 15, Men(%) = 53, Age = 43.4 ± 12.49 yrs, Hb(g/dL) = 9.05 ± 0.97, Ferritin (mg/dL) = 303.34 ± 120.41, TIBC = 269 ± 94.59, Serum iron (mg/dL) = 92.62 ± 36.73	12 weeks	Pentoxifylline 400 mg/day (n = 15)	Hemoglobin (g/dL)	Significant increase from 9.053 ± 0.976 to 9.980 ± 1.991 (p = 0.02)

Legend: CKD: chronic kidney disease, DM: diabetes mellitus, ESA: erythropoiesis-stimulating agent, ESKD: end stage kidney disease, Hb: hemoglobin, HD: hemodialysis, IU: international units, PTX: pentoxifylline, TIBC: Total Iron Binding Capacity, TSAT: transferrin saturation.

**Table 2:** Summary of the main characteristics of the randomized studies reviewed.

Study, year (ref)	Study population	Population characteristics	Duration	Intervention	Comparator	Outcome(s)	Results
Navarro et al. 1999 [18]	Anemic patients with advanced CKD (C_Cr_<30 mL/min), no clinical evidence of blood losses and normal iron status (serum ferritin>50 ng/mL and TSAT>20%)	N = 12, Men(%) = 58, Age = 65±7 yrs, Hb (g/dL) = 10±0.6, Ferritin (ng/mL) = 84±12, TSAT(%) = 28±4, C_Cr_(mL/min) = 25±4	6 months	Pentoxifylline 400 mg/day (n = 7)	Placebo (n = 5)	Hemoglobin (g/dL)	-Significant increase (from 9.9 ±0.5 to 10.6±0.6, p<0.01) in PTX group-No changes in controls
						Hematocrit (%)	-Significant increase (from 27.9 ±1.6 to 31.3±1.9, p<0.01) in PTX group-No changes in controls
Perkins et al. 2009 [19]	Hypertensive patients (age>18 yrs) with decreased GFR and proteinuria >1 g/24h treated with RAS blockers	N = 39, Men(%) = 62, Age = 64.3 yrs, Hct (%) = 34.3	12 months	Pentoxifylline 400 mg/twice a day (n = 22)	Placebo (n = 17)	Hematocrit (%)	No difference between groups at the end of the study (33±5.9 vs. 34.1±4.9 in PTX vs. PLB, p = 0.3)
Gonzalez-Espinoza et al. 2012 [20]	Patients on chronic HD for at least 2 months, age ≥18 yrs	N = 36, Men(%) = 69, Age = 35.3 yrs	4 months	Pentoxifylline 400 mg/ day (n = 18)	Placebo (n = 18)	ESA dose (IU/Kg)	No difference between groups [50 (0–60) vs. 50 (0–60)] and within groups [50 (0–60) vs. 50 (0–60) and 50 (47–60) vs. 50 (0–60) in PTX and PLB respectively, p = NS] at the end of the study
						Hemoglobin (g/dL)	No difference between groups (9.6±1.9 vs. 10.5±2.7) and within groups (10.6±1.8 vs 9.6±1.9 and 10.7±2.1 vs. 10.5±2.7 in PTX and PLB respectively, p = NS) at the end of study
Mortazavi et al. 2012 [21]	Patients on chronic HD patients for at least 1 month with Hb <10.7 g/dL at least 2 times	N = 50, Men(%) = 66, Age = 57.2yrs	6 months	Pentoxifylline 400 mg/a day (n = 25)	Placebo (n = 25)	Hemoglobin(g/dL)	No changes in both groups
						Serum iron (mg/dL)	Changes at the end of the study were significantly different between groups (-62±56.8 vs -13.2±47.1 in PTX vs PLB, p = 0.005)
						TIBC	No difference in changes between groups
						Ferritin (ng/mL)	No difference in changes between groups
						ESA dose (UI/Kg)	No difference in changes between groups
AIONID 2013 [22]	Chronic HD patients with serum albumin <4.0 g/dL persisting for at least 3 months	N = 43, Men(%) = 58, Age = 61yrs, DM(%) = 67	16 weeks	Pentoxifylline 400 mg/day (n = 22)	Placebo (n = 21)	Hemoglobin (g/dL)	Changes at the end of the study were not significantly different between groups
						Ferritin (ng/mL)	-Increase from 464±301 to 789±396 in PTX group-Decrease from 673±304 to 640±357 in PLB group
						TSAT (%)	-Increase from 26±12 to 32±9 in PTX group-Increase from 32±16 to 35±22 in PLB group
Antunes et al. 2014 [23]	Chronic HD patients with CRP≥0.5 mg/dL in screening test	N = 71, Men (%) = 64,Age = 55.7 yrs, DM(%) = 20, Hb (mg/dL) = 10.95, Ferritin (ng/dL) = 328.5, TSAT(%) = 31.4, Hepcidin (ng/mL) = 127.7	3 months	Pentoxifylline (400mg/thrice weekly) (n = 35)	Standard therapy (n = 36)	Hemoglobin (g/dL)	No difference between groups at the end of the study
						TSAT (%)	No difference between groups at the end of the study
						Hepcidin (ng/mL)	No difference between groups at the end of the study
HERO 2015 [24]	Patients with CKD stage 4–5, (including HD) with ESA-hyporesponsive anemia (hemoglobin ≤120 g/L and ESA resistance index ≥1.0 IU/kg/wk/g/L for erythropoietin-treated patients and ≥0.005 mg/kg/wk/g/L for darbepoetin-treated patients)	N = 53, Men(%) = 45, Age = 62.1 yrs, DM(%) = 47, Hb (g/L) = 106, Serum ferritin (mcg/L) = 486.5, ESA dose (IU/kg/wk) = 236, ERI (IU/kg/wk/g/L) = 2.37	4 months	Pentoxifylline (400 mg/day) (n = 26)	Placebo (n = 27)	ERI (IU/kg/wk/g/L)	No difference between groups (data adjusted for baseline values)
						Hemoglobin (g/L)	Significant differences between groups (110.9 vs 103.2 in PTX vs PLB, p = 0.01; data adjusted for baseline values)
						ESA dose (IU/kg/wk)	No difference between groups (data adjusted for baseline values)
						Serum ferritin (mcg/L)	No difference between groups (data adjusted for baseline values)
						TSAT (%)	No difference between groups (data adjusted for baseline values)

Legend: CCr: creatinine clearance, CKD: chronic kidney disease, CRP: C-reactive protein, DM: diabetes mellitus, ERI: erythropoiesis-stimulating agent resistance index, ESA: erythropoiesis-stimulating agent, GFR: glomerular filtration rate, Hb: hemoglobin, Hct: hematocrit, HD: hemodialysis, IU: international units, PLB: placebo, PTX: pentoxifylline, RAS: Renin-Angiotensin-System, TIBC: Total Iron Binding Capacity, TSAT: transferrin saturation.

### Study characteristics

Amongst the 11 studies reviewed there were seven randomized controlled trials [18–24], one retrospective case-control study [16] and three prospective uncontrolled studies [14, 15, 17]. All studies but one [24] were single center. The final population analyzed in this review included 377 patients but the range was variable across studies, spanning from ten [15] to 71 [23]. Most studies included ESKD patients on chronic renal replacement therapy [14, 16, 17, 20–24]. Five studies analyzed patients on CKD on conservative therapy with various degree of renal function impairment [14, 15, 18, 19, 24].

Five RCTs compared pentoxifylline to placebo [18–21, 24] while in the RCT by Antunes [23] the control group was on standard therapy. The AIONID trial [22] compared the effect of three types of interventions (pentoxifylline, oral nutrition supplements combined to anti-inflammatory and anti-oxidant products- ONS- and pentoxifylline plus ONS) versus placebo in 84 dialysis patients. To maximize consistency with the interventions adopted in other studies only data from the pentoxifylline (n = 22) and placebo arms (n = 21) were used in pooled analyses.

Patients received pentoxifylline at the daily dose of 400 mg in the vast majority of studies [14, 15, 17, 18, 20–22, 24]; in two studies [16, 19] 400 mg of pentoxifylline were administered twice a day; in only one study [23] pentoxifylline was administered 400 mg/thrice weekly. In five studies [14, 17, 20, 23, 24] patients were also treated with ESAs. Three studies declared that patients were also treated with iron supplements [20, 23, 24]. However, only in one study [23] detailed information about dose regimen was provided (spanning from 200 to ≥400 mg/month).Three studies included by protocol patients with documented hypo-responsiveness to ESAs [14, 17, 24]. Male gender spanned from 45% [24] to 69% [20]. Information on diabetes mellitus was provided in four studies [16, 22–24] and this condition was present in a percentage of the study populations ranging from 14 [16] to 67% [22].

Study duration varied from four weeks [15] to 32 months [16].

### Study quality and risk of bias

Risk of bias in RCTs is summarized in S2 Table. Random sequence generation was detailed in five trials [18–20, 22, 24] and allocation concealment in three [20, 22, 24]. Two RCTs were double blind [19, 22], two were triple blind [20, 24] and one study was open label [23]. In two studies [18, 21], blinding of participants, investigators and outcome assessors was not specified. The HERO trial [24] might be considered at high risk of attrition bias. The overall drop-out rate in this study was 16.9% but patients who discontinued the study were unbalanced between the active and control arm (23 vs. 11%) and data were analyzed on an intention-to-treat basis. Risk of attrition bias was high also in the AIONID trial [22] as 21% of the whole population discontinued the trial. In three other RCTs such risk was low [18–20]. Attrition bias was not assessable in two studies as the drop-out rate was not specified [21, 23]. Reporting bias was low in four trials [19, 20, 22, 24] and unclear in the remainder.

Risk of confounding by indication was apparently high in all non-randomized studies as patients were selected for pentoxifylline treatment according to the presence of anemia resistant to ESAs or iron therapy. In one retrospective case-control study [16], cases were represented by patients already on pentoxifylline treatment for peripheral vascular disease.

### Outcome data

End of treatment data on hemoglobin were available in six RCTs [18, 20–24], three prospective uncontrolled [14, 15, 17] and one retrospective case-control study [16]. Data on hematocrit variation were reported in two RCTs [18, 19]. Information on ESAs dosage in patients receiving or not receiving pentoxifylline was recorded in three RCTs [20, 21, 24] and one retrospective, case-control study [16], while changes in the ESA resistance index were analyzed only in a single trial [24]. Variations in TSAT were provided in three RCTs [22–24] and one prospective uncontrolled study [15] while those in serum ferritin were available in three RCTs [21, 22, 24], one prospective uncontrolled [15] and one retrospective study [16]. Data on total serum iron and total iron binding capacity were available only in one study [21], as well as data on end of treatment hepcidin [23]. Nine studies [14–16, 19–24] looked by protocol at possible side effects related to treatment with five [14, 19, 22–24] reporting adverse events requiring treatment discontinuation or medical intervention (Table 3).

**Table 3:** Main adverse events recorded in the reviewed studies.

Study	Event	Incidence by group (n/pts) PTX vs. Control	
Cooper et al. 2004 [14]	Nausea	1/12	
	Confusion	1/12	
Ferrari et al. 2010 [15]	No events	-	
Mora-Gutiérrez et al. 2013[16]	No events	-	-
Gonzalez-Espinoza et al. 2012 [20]	No events	-	-
Mortazavi et al. 2012 [21]	No events	-	-
Perkins et al. 2009 [19]	Mild tremors	1/22	0/17
AIONID 2013 [22]	Gastrointestinal	1/22	2/21
Antunes et al. 2014 [23]	Gastrointestinal	2/35	0/36
HERO 2015 [24]	Cardiovascular	1/26	2/27
	Central nervous system	1/26	0/27
	Gastrointestinal	0/26	2/27
	Hematologic	1/26	1/27
	Hepatic	0/26	1/27
	Musculoskeletal	4/26	0/27
	Renal dialysis	2/26	9/27
	Respiratory	2/26	1/27

Legend: PTX: pentoxifylline

### Effects of pentoxifylline on primary and secondary outcomes

#### Hemoglobin

**Fig 2:**
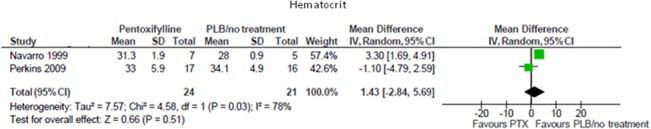
Effects of pentoxifylline vs. placebo/standard treatment on hemoglobin levels.

In three small, prospective uncontrolled studies [14, 15, 17] including a total of 37 patients, pentoxifylline significantly increased hemoglobin from pre-treatment levels (Table 1). Conversely, in a pooled meta-analysis of seven controlled studies (299 participants), pentoxifylline did not produce significant changes on hemoglobin levels when compared with placebo or standard therapy (MD 0.12 g/dL; 95% CI -0.22, 0.47; Fig 2), with low evidence for heterogeneity in the analysis (Chi² = 9.58, p = 0.14; I² = 37%).

#### Hematocrit

**Fig 3:**
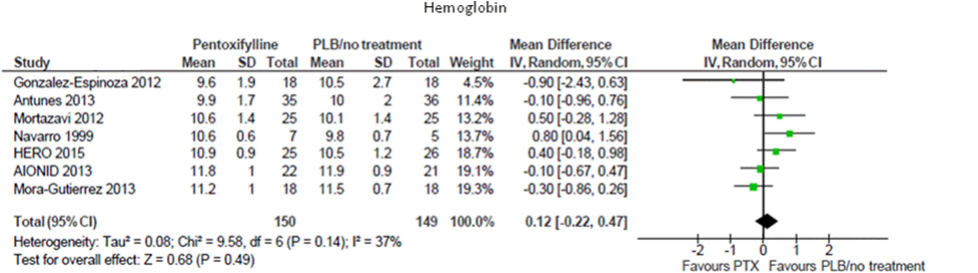
Effects of pentoxifylline vs. placebo/standard treatment on hematocrit.

In data pooled from two RCTs (45 participants), pentoxifylline was not superior to placebo/standard therapy in improving hematocrit levels (MD 1.43%; 95% CI -2.84, 5.69; Fig 3). There was significant heterogeneity in this analysis (Chi² = 4.58, p = 0.03; I² = 78%) that could not be further explored as only two studies were included.

#### ESAs-dosage

**Fig 4:**
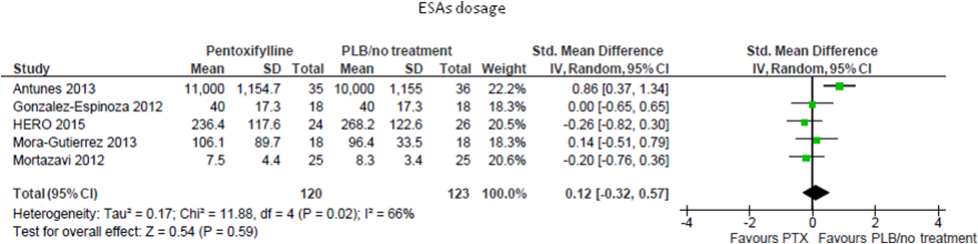
Effects of pentoxifylline vs. placebo/standard treatment on ESAs dosage.

In a pooled meta-analysis including five studies (243 participants), pentoxifylline had no tangible effects over placebo/standard treatment on the needed dose of ESAs (SMD 0.12; 95%CI -0.32, 0.57; Fig 4). This analysis was affected by high heterogeneity (Chi² = 11.88, p = 0.02; I² = 66%) that was fully explained by the sole RCT [23] in which pentoxifylline was administered on a thrice/weekly- rather than a daily-regimen.

#### ESA resistance (ERI)

Information on the resistance to ESA was available in only one trial [24], reporting no differences at the end of the study between the pentoxifylline and the placebo group after adjustment for baseline values (2.21 vs 2.60 IU/kg/wk/g/L, p = 0.1)

#### Iron indexes

**Fig 5:**
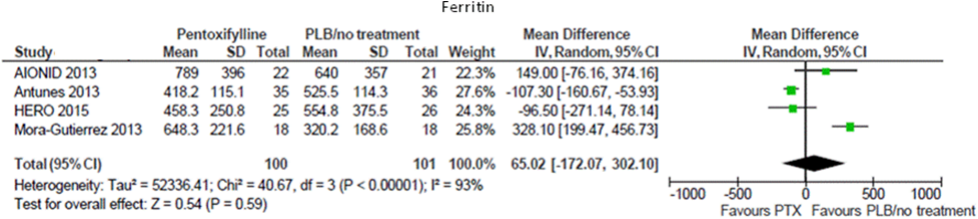
Effects of pentoxifylline vs. placebo/standard treatment on ferritin.

Serum ferritin values remained unchanged after pentoxifylline treatment in one prospective uncontrolled study [15]. This observation was consistent with a meta-analysis of four controlled studies (201 participants), showing no difference in end of treatment ferritin between subjects receiving pentoxifylline or placebo/standard therapy (MD 65.02 mg/L; 95% CI -172.07, 302.10; Fig 5). There was high heterogeneity in this analysis (Chi² = 40.67, p<0.00001; I² = 93%) that was significantly reduced (I² = 58%) after excluding data from the only retrospective study [16].

**Fig 6:**
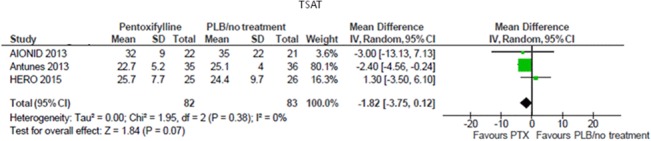
Effects of pentoxifylline vs. placebo/standard treatment on TSAT.

In a single prospective uncontrolled study [15], 1-month therapy with pentoxifylline significantly increased TSAT values (13± 3 to 20±5%, p<0.003) in ten patients. In a meta-analysis pooling data from three RCTs (165 participants), pentoxifylline did not affect TSAT levels as compared with placebo/standard therapy (MD -1.82%; 95% CI -3.75, 0.12; Fig 6), with no heterogeneity in the analysis (Chi² = 1.95, p = 0.38; I² = 0%).

Data on total serum iron and total iron binding capacity (TIBC) were available in a single RCT [21]; changes in serum iron were significantly different between the pentoxifylline and placebo groups (-62±56.8 vs. -13.2±47.1, p = 0.005), conversely, there was no difference with respect to TIBC (-37±63.4 vs. -34±55.5; p = 0.87).

In one trial [23], end of study hepcidin levels were similar in patients treated with pentoxifylline or standard therapy (77.6 (62.2–88) vs. 70.5 (54.9–89.1); p = 0.45).

#### Adverse events

**Fig 7:**
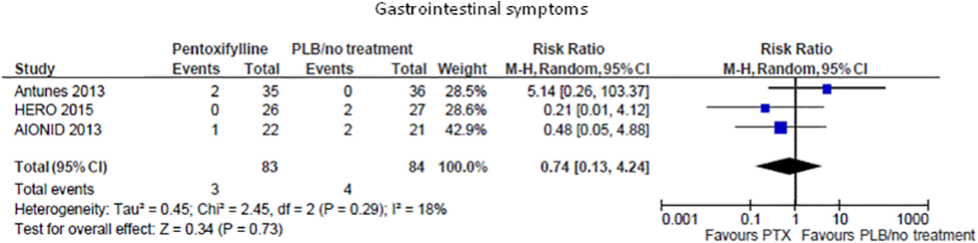
Risk of gastrointestinal symptoms in pentoxifylline vs. placebo/standard treatment.

Two uncontrolled [14, 15] and seven controlled studies [16, 19–24] looked at possible side effects related to treatment (Table 2). In four studies [15, 16, 20, 21] no major adverse events were apparently reported in the whole study population. In one uncontrolled study [14] pentoxifylline treatment was associated with nausea and confusion in one patient apiece. In one RCT [19] one patient in the active group experienced mild tremors. In the HERO study [24] several side effects were recorded during the trial but their occurrence was not statistically different between the pentoxifylline and the placebo arms. In a pooled meta-analysis from three RCTs (167 participants) pentoxifylline was not associated with a higher risk of gastrointestinal symptoms than placebo/standard therapy (RR 0.74; 95%CI 0.13, 4.24; Fig 7) with low heterogeneity in the analysis (Chi² = 2.45,p = 0.29; I² = 18%).

#### Subgroup analyses

Given the overall paucity of studies looking at similar outcomes, we could not perform reliable subgroup analyses according to study design, duration of follow-up, administered dose of pentoxifylline, population characteristics, baseline hemoglobin levels and iron status, concomitant treatment with ESAs or iron supplements, as originally planned in the review protocol.

## Discussion

The main aim of this review was to ascertain whether pentoxifylline supplements might be useful for improving anemia control in chronic kidney disease.

Results from our systematic literature search indicates that the evidence on this issue relies on few uncontrolled, low-quality studies and some randomized controlled trials (all comparing pentoxifylline treatment to placebo or standard therapy) with sufficient study quality but small sample size. Although providing information on biomarkers pertinent with the outcome of interest, some of these studies were not primarily designed to investigate the effect of pentoxifylline on anemia. The small sample size of such studies could be therefore not adequately powered to catch a significant treatment effect on certain biomarkers. There was also high heterogeneity among studies with respect to duration of follow-up, CKD and anemia severity, background therapy or responsiveness to ESAs or iron.

Inflammation plays a pivotal role in the genesis and worsening of anemic states secondary to various diseases. This holds true particularly in CKD. Uremic patients are in a perpetual state of both immunosuppression and chronic activation of the immune system [25].

**Fig 8:**
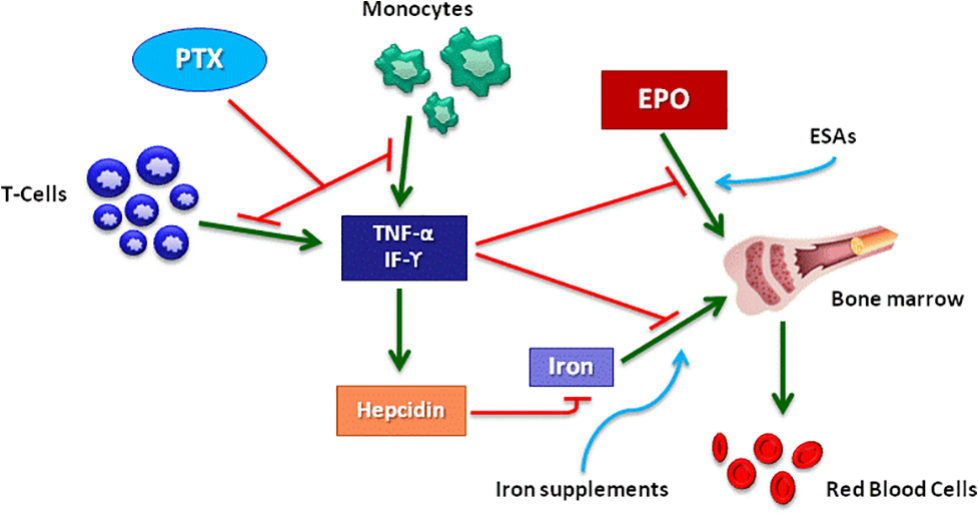
Inflammation plays a key-role in the genesis of anemia in CKD. TNF-α and IF-ϒ are pro-inflammatory cytokines which may generate or worsen anemia by limiting the pro-erythropoietic effect of EPO and ESAs at the medullary level. In addition, such cytokines may reduce iron bio-availability by increasing serum hepcidin levels. Pentoxifylline would improve anemia in CKD by inhibiting the release of TNF-α and IF-ϒ from monocytes and T-cells. This would ameliorate the medullary sensitivity to endogenous EPO and exogenous ESAs and reduce the need for iron supplements by increasing endogenous iron availability. Legend to Fig 8: PTX: pentoxifylline, TNF-α: tumor necrosis factor alpha, EPO: erythropoietin, ESAs: erythropoiesis stimulating agents, IF-ϒ: interferon gamma

Pentoxifylline might be considered as a non-specific approach for limiting the effect of inflammation on anemia in CKD (Fig 8). Indeed, in addition to anti-platelet and hemorheological properties, this agent is endowed with inhibitory actions on TNF-α production from monocytes and TNF-α and IF-ϒ release from T-cells. Benefits related to TNF-α blockade have already been demonstrated in other pathological conditions, such as acute shocks, rheumatoid arthritis, systemic vasculitis, idiopathic dilated cardiomyopathy and type-1 diabetes mellitus [26].

Although evidence exists that pentoxifylline administration may reduce inflammation in CKD patients [27], the question as to whether such effect may translate into an improvement in anemia control remains debated. Pentoxifylline treatment was able to increase significantly hemoglobin levels in four non-randomized studies [14–17], of which three were uncontrolled, although the possible influence of selection, observer and co-intervention biases cannot be fully ruled out. Hemoglobin was significantly improved in one randomized trial [18] which, however, included only patients with mild anemia and normal iron parameters. In the HERO trial [24] a difference in end of study hemoglobin was evidenced between the treated and the untreated group but such difference was significant only after adjustment for baseline values. These single, positive observations were not confirmed in our cumulative meta-analysis of seven controlled studies which showed no substantial evidence of benefits of pentoxifylline on hemoglobin as compared to standard care. Nevertheless these findings, although negative, should always be weighed against the clinical and methodological diversity, the heterogeneity observed among the single trials, the overall poor study quality and the absence of focus on ESA hypo-responsiveness. Similarly, no definite conclusion can be drawn on hematocrit levels.

Sustained inflammation might trigger or worsen anemia through additional mechanisms that reduce iron balance and bio-availability. Nevertheless, the vast majority of studies included in our review did not report significant changes in any iron parameter after treatment with pentoxifylline. TSAT and total serum iron were somewhat improved only in one prospective uncontrolled study [15] and in one RCT [21], respectively. Interestingly, pentoxifylline administration had no consequence on serum hepcidin levels in a RCT of hemodialysis patients specifically designed for investigating such possible effect [23]. This was confirmed in our pooled meta-analyses showing no changes in ferritin, TSAT and total serum iron in patients receiving pentoxifylline rather than standard therapy or placebo.

Two more issues are worth to be addressed. First, all the studies reviewed provided information on the effects of pentoxifylline on biomarkers of anemia. However, these biomarkers must be considered as surrogate rather than primary endpoints. Poor anemia control in CKD is associated with worse cardiovascular outcomes and reduced quality of life, particularly in dialysis patients [28]. Hence, trials testing emerging therapies must clarify whether possible improvements in anemia parameters actually translate into real clinical benefits in the long-term. Among the studies reviewed, only the HERO trial [24] provided information on the effect of pentoxifylline treatment on quality of life, showing no significant differences over placebo at study end. Conversely, there were no trials focusing on cardiovascular outcomes such as mortality or non-fatal cardiovascular events, probably due to the difficulty of recruiting larger study populations and due to the necessity of longer treatment periods. Interestingly, benefits of this agent on these hard endpoints have already been demonstrated in other clinical settings, such as chronic heart failure [29] or acute sepsis in newborns [30]. Second, as briefly alluded to before, the target population which would benefit the most from pentoxifylline has not yet been well defined. Anemia management in ESKD patients on chronic dialysis is notoriously more challenging than in early CKD stages, mostly due to the increasing co-morbidity and the more elevated levels of inflammation [31, 32]. Hypo-responsiveness to ESAs, that is pervasive in dialysis patients, often demands for intravenous iron supplements or increasing doses of these agents. Higher doses of ESAs have been associated with an increased risk of death, adverse cardiovascular events, stroke and malignancy [4, 5]. Alternative treatments able to reduce ESAs need would therefore provide clinical benefits beyond cost-saving. Despite low-quality evidence suggested the potential usefulness of pentoxifylline supplements in this respect [16], in our meta-analysis of multiple trials there was no proof of concrete benefits of this agent on the dose of ESAs needed. Furthermore, in the only trial providing data on ESAs resistance [24], pentoxifylline was not superior to placebo in improving this parameter after adjustment for baseline values.

Our review has some points of strength and limitations that deserve mentioning.

Strengths include a pre-published protocol, a thorough systematic search of different medical databases to minimize publication bias, data extraction and analysis and trial quality assessment by two independent reviewers according to current methodology standards. The main limitations of this review are mostly represented by the exclusive search for English-language articles and the number and quality of data available from studies. The majority of the included studies had a small sample size and there were few studies providing information on similar outcomes. This may limit the reliability of findings from pooled meta-analyses and prevented us to perform exhaustive subgroup analyses for identifying effect modifiers as originally planned. Furthermore, the above mentioned heterogeneity in the study populations, in particular with respect to CKD and anemia severity, duration of intervention and responsiveness/current therapy with iron or ESAs, prevented drawing definite conclusions and hampered the generalizability of findings to the whole CKD population.

In conclusion, to date, there is no substantial evidence supporting the utility of pentoxifylline for improving anemia control in CKD patients. However, this deduction relies on few small and heterogeneous studies looking at surrogate biomarkers. Future trials adequately powered on hard, patient-centered endpoints are advocated to clarify the potential role of pentoxifylline supplements for improving anemia-related outcomes in the CKD population.

## Supporting Information

S1 Checklist PRISMA checklist of this systematic review. (DOC)

S1 Table Search strategy in CENTRAL, Ovid-Medline, EMBASE and PubMed databases. (DOCX)

S2 Table Risk of bias in randomized controlled trials. (DOCX)

## References

[1] Stauffer ME, Fan T. Prevalence of anemia in chronic kidney disease in the United States. PloS one. 2014;9(1):e84943 10.1371/journal.pone.0084943 24392162 PMC3879360

[2] Tonelli M, Winkelmayer WC, Jindal KK, Owen WF, Manns BJ. The cost-effectiveness of maintaining higher hemoglobin targets with erythropoietin in hemodialysis patients. Kidney Int. 2003;64(1):295–304. 10.1046/j.1523-1755.2003.00079.x . 12787422

[3] Clement FM, Klarenbach S, Tonelli M, Wiebe N, Hemmelgarn B, Manns BJ. An economic evaluation of erythropoiesis-stimulating agents in CKD. American journal of kidney diseases: the official journal of the National Kidney Foundation. 2010;56(6):1050–61. 10.1053/j.ajkd.2010.07.015 . 20932621

[4] Szczech LA, Barnhart HX, Inrig JK, Reddan DN, Sapp S, Califf RM, et al Secondary analysis of the CHOIR trial epoetin-alpha dose and achieved hemoglobin outcomes. Kidney Int. 2008;74(6):791–8. 10.1038/ki.2008.295 18596733 PMC2902279

[5] Solomon SD, Uno H, Lewis EF, Eckardt KU, Lin J, Burdmann EA, et al Erythropoietic response and outcomes in kidney disease and type 2 diabetes. The New England journal of medicine. 2010;363(12):1146–55. 10.1056/NEJMoa1005109 . 20843249

[6] Jennings DL, Williams CT, Morgan JA. Pentoxifylline for the treatment of hemolytic anemia in a patient who developed recurrent gastrointestinal bleeding while on continuous-flow left ventricular assist device support. ASAIO journal. 2013;59(5):526–7. 10.1097/MAT.0b013e31829f0eb1 . 23896772

[7] Assem M, Yousri M. Impact of pentoxifylline and vitamin e on ribavirin-induced haemolytic anaemia in chronic hepatitis C patients: an egyptian survey. International journal of hepatology. 2011;2011:530949 10.4061/2011/530949 21994862 PMC3170830

[8] Raza A, Qawi H, Andric T, Dar S, Lisak L, Huang RW, et al Pentoxifylline, Ciprofloxacin and Dexamethasone Improve the Ineffective Hematopoiesis in Myelodysplastic Syndrome Patients; Malignancy. Hematology. 2000;5(4):275–84. . 11399622 10.1080/10245332.2000.11746517

[9] Sherer JT, Glover PH. Pentoxifylline for sickle-cell disease. The Annals of pharmacotherapy. 2000;34(9):1070–4. . 10981255 10.1345/aph.19397

[10] Rho M. Do pentoxifylline and ascorbic acid improve erythropoiesis stimulating agent resistance? Semin Dial. 2011;24(4):378–9. Epub 2011/08/02. 10.1111/j.1525-139X.2011.00894.x . 21801213

[11] National Kidney F. K/DOQI clinical practice guidelines for chronic kidney disease: evaluation, classification, and stratification. American journal of kidney diseases: the official journal of the National Kidney Foundation. 2002;39(2 Suppl 1):S1–266. . 11904577

[12] Hozo SP, Djulbegovic B, Hozo I. Estimating the mean and variance from the median, range, and the size of a sample. BMC medical research methodology. 2005;5:13 10.1186/1471-2288-5-13 15840177 PMC1097734

[13] Higgins JP, Thompson SG, Deeks JJ, Altman DG. Measuring inconsistency in meta-analyses. Bmj. 2003;327(7414):557–60. 10.1136/bmj.327.7414.557 12958120 PMC192859

[14] Cooper A, Mikhail A, Lethbridge MW, Kemeny DM, Macdougall IC. Pentoxifylline improves hemoglobin levels in patients with erythropoietin-resistant anemia in renal failure. Journal of the American Society of Nephrology. 2004;15(7):1877–82. . 15213276 10.1097/01.asn.0000131523.17045.56

[15] Ferrari P, Mallon D, Trinder D, Olynyk JK. Pentoxifylline improves haemoglobin and interleukin-6 levels in chronic kidney disease. Nephrology (Carlton, Vic). 2010;15(3):344–9. Epub 2010/05/18. 10.1111/j.1440-1797.2009.01203.x . 20470305

[16] Mora-Gutierrez JM, Ferrer-Nadal A, Garcia-Fernandez N. Effect of pentoxifylline on anaemia control in haemodialysis patients: retrospective observational case-control study. Nefrologia. 2013;33(4):524–31. Epub 2013/07/31. 10.3265/Nefrologia.pre2013.Apr.11654 . 23897184

[17] Mohammadpour AH, Nazemian F, Khaiat MH, Tafaghodi M, Salari P, Charkazi S, et al Evaluation of the effect of pentoxifylline on erythropoietin-resistant anemia in hemodialysis patients. Saudi journal of kidney diseases and transplantation: an official publication of the Saudi Center for Organ Transplantation, Saudi Arabia. 2014;25(1):73–8. Epub 2014/01/18. . 24434385 10.4103/1319-2442.124492

[18] Navarro JF, Mora C, Garcia J, Rivero A, Macia M, Gallego E, et al Effects of pentoxifylline on the haematologic status in anaemic patients with advanced renal failure. Scandinavian journal of urology and nephrology. 1999;33(2):121–5. Epub 1999/06/09. . 10360454 10.1080/003655999750016113

[19] Perkins RM, Aboudara MC, Uy AL, Olson SW, Cushner HM, Yuan CM. Effect of pentoxifylline on GFR decline in CKD: a pilot, double-blind, randomized, placebo-controlled trial. American journal of kidney diseases: the official journal of the National Kidney Foundation. 2009;53(4):606–16. Epub 2009/02/14. 10.1053/j.ajkd.2008.11.026 . 19216016

[20] González-Espinoza L, Rojas-Campos E, Medina-Pérez M, Peña-Quintero P, Gómez-Navarro B, Cueto-Manzano AM. Pentoxifylline decreases serum levels of tumor necrosis factor alpha, interleukin 6 and C-reactive protein in hemodialysis patients: results of a randomized double-blind, controlled clinical trial. Nephrology, dialysis, transplantation [Internet]. 2012; 27(5):[2023–8 pp.]. Available from: http://onlinelibrary.wiley.com/o/cochrane/clcentral/articles/324/CN-00882324/frame.html. 10.1093/ndt/gfr579 21968012

[21] Mortazavi M, Seyrafian S, Taheri S, Nasiri R, Dolatkhah S, Naini AE, et al Role of pentoxifylline in treatment of anemic patients suffering chronic hemodialysis: a randomized clinical trial. Med Arh. 2012;66(2):84–6. Epub 2012/04/11. . 22486134 10.5455/medarh.2012.66.84-86

[22] Rattanasompattikul M, Molnar MZ, Lee ML, Dukkipati R, Bross R, Jing J, et al Anti-Inflammatory and Anti-Oxidative Nutrition in Hypoalbuminemic Dialysis Patients (AIONID) study: results of the pilot-feasibility, double-blind, randomized, placebo-controlled trial. J Cachexia Sarcopenia Muscle. 2013;4(4):247–57. Epub 2013/09/21. 10.1007/s13539-013-0115-9 ; PubMed Central PMCID: PMCPmc3830006. 24052226 PMC3830006

[23] Antunes SA, Vilela RQ, Vaz JD, Canziani MEF. Pentoxifylline does not alter the concentration of hepcidin in chronic kidney disease patients undergoing hemodialysis. International journal of artificial organs [Internet]. 2014; 37(7):[521–8 pp.]. Available from: http://onlinelibrary.wiley.com/o/cochrane/clcentral/articles/416/CN-01041416/frame.html. 10.5301/ijao.5000340 25044383

[24] Johnson DW, Pascoe EM, Badve SV, Dalziel K, Cass A, Clarke P, et al A randomized, placebo-controlled trial of pentoxifylline on erythropoiesis-stimulating agent hyporesponsiveness in anemic patients with CKD: the Handling Erythropoietin Resistance With Oxpentifylline (HERO) trial. American journal of kidney diseases: the official journal of the National Kidney Foundation. 2015;65(1):49–57. Epub 2014/08/15. 10.1053/j.ajkd.2014.06.020 . 25115616

[25] Betjes MG. Immune cell dysfunction and inflammation in end-stage renal disease. Nature reviews Nephrology. 2013;9(5):255–65. 10.1038/nrneph.2013.44 . 23507826

[26] Kreth S, Ledderose C, Luchting B, Weis F, Thiel M. Immunomodulatory properties of pentoxifylline are mediated via adenosine-dependent pathways. Shock. 2010;34(1):10–6. 10.1097/SHK.0b013e3181cdc3e2 . 19997047

[27] Vilayur E, Harris DC. Emerging therapies for chronic kidney disease: what is their role? Nature reviews Nephrology. 2009;5(7):375–83. 10.1038/nrneph.2009.76 . 19455178

[28] Horl WH. Anaemia management and mortality risk in chronic kidney disease. Nature reviews Nephrology. 2013;9(5):291–301. 10.1038/nrneph.2013.21 . 23438972

[29] Champion S, Lapidus N, Cherie G, Spagnoli V, Oliary J, Solal AC. Pentoxifylline in heart failure: a meta-analysis of clinical trials. Cardiovascular therapeutics. 2014;32(4):159–62. 10.1111/1755-5922.12076 . 24758396

[30] Pammi M, Haque KN. Pentoxifylline for treatment of sepsis and necrotizing enterocolitis in neonates. The Cochrane database of systematic reviews. 2015;3:CD004205 10.1002/14651858.CD004205.pub3 . 25751631

[31] Babitt JL, Lin HY. Mechanisms of anemia in CKD. J Am Soc Nephrol. 2012;23(10):1631–4. 10.1681/ASN.2011111078 22935483 PMC3458456

[32] Cooper AC, Mikhail A, Lethbridge MW, Kemeny DM, Macdougall IC. Increased expression of erythropoiesis inhibiting cytokines (IFN-gamma, TNF-alpha, IL-10, and IL-13) by T cells in patients exhibiting a poor response to erythropoietin therapy. J Am Soc Nephrol. 2003;14(7):1776–84. . 12819237 10.1097/01.asn.0000071514.36428.61

