# Supplementary material for: Pentoxifylline for Anemia in Chronic Kidney Disease: A Systematic Review and Meta-Analysis
Source: PLoS One. 2015 Aug 3;10(8):e0134104. doi: 10.1371/journal.pone.0134104 (PMC4523191; doi:10.1371/journal.pone.0134104)
Supplement: S1 Table — (DOCX) [file pone.0134104.s002.docx]

**Supplementary Table S1**. Search strategy in CENTRAL, Ovid-Medline, EMBASE and PubMed databases

| **CENTRAL** |
| --- |
| #1 dialysis:ti,ab,kw  #2 (hemofiltration or haemofiltration):ti,ab,kw  #3 (hemodiafiltration or haemodiafiltration):ti,ab,kw  #4 (end-stage renal or end-stage kidney or endstage renal or endstage kidney):ti,ab,kw  #5 (ESRF or ESKF or ESRD or ESKD):ti,ab,kw  #6 (chronic kidney or chronic renal):ti,ab,kw  #7 (CKF or CKD or CRF or CRD):ti,ab,kw  #8 (CAPD or CCPD or APD):ti,ab,kw  #9 (predialysis or pre-dialysis):ti,ab,kw  #10 MeSH descriptor Kidney Failure, Chronic explode all trees  #11 MeSH descriptor Renal Replacement Therapy explode all trees  #12 MeSH descriptor Renal Insufficiency, Chronic explode all trees  #13 (#1 OR #2 OR #3 OR #4 OR #5 OR #6 OR #7 OR #8 OR #9 OR #10 OR #11 OR #12)  #14 MeSH descriptor Pentoxifylline, this term only  #15 (oxipentifylline):ti,ab.kw  #16 (pentoxifylline):ti,ab,kw  #17 (trental):ti,ab,kw  #18 (#15 OR #16 OR #17)  #19 (#13 AND #18) |
| **OVID-Medline** |
| 1. exp Renal Dialysis/  2. (hemodialysis or haemodialysis).tw.  3. (hemofiltration or haemofiltration).tw.  4. (hemodiafiltration or haemodiafiltration).tw.  5. dialysis.tw.  6. (CAPD or CCPD or APD).tw.  7. Renal Insufficiency/  8. Kidney Failure/  9. exp Renal Insufficiency, Chronic/  10. Kidney Diseases/  11. Uremia/  12. (end-stage renal or end-stage kidney or endstage renal or endstage kidney).tw.  13. (ESRF or ESKF or ESRD or ESKD).tw.  14. (chronic kidney or chronic renal).tw.  15. (CKF or CKD or CRF or CRD).tw.  16. (predialysis or pre-dialysis).tw.  17. ur?emi$.tw.  18. or/1-17  19. exp Pentoxifylline/  20. oxipentifylline.tw  21. pentoxifylline.tw  22. trental.tw  23. or/19-22  24. 18 and 23 |
| **EMBASE** |
| 1. PENTOXIFYLLINE  2. pentoxifylline.tw.  3. oxpentifylline.tw.  4. trental.tw.  5. torental.tw.  6. BL-191.tw.  7. agapurin.tw.  8. or/1-7  9. exp Renal Replacement Therapy/  10. (hemodialysis or haemodialysis).tw.  11. (hemofiltration or haemofiltration).tw.  12. (hemodiafiltration or haemodiafiltration).tw.  13. dialysis.tw.  14. (CAPD or CCPD or APD).tw.  15. Kidney Disease/  16. Chronic Kidney Disease/  17. Kidney Failure/  18. Chronic Kidney Failure/  19. Uremia/  20. (chronic kidney or chronic renal).tw.  21. (CKF or CKD or CRF or CRD).tw.  22. (end-stage renal or end-stage kidney or endstage renal or endstage kidney).tw.  23. (ESRF or ESKF or ESRD or ESKD).tw.  24. (predialysis or pre-dialysis).tw.  25. ur?emi$.tw.  26. or/9-25  27. 8 and 26 |
| **PubMed** |
| ((chronic kidney disease OR CKD OR chronic renal failure OR chronic renal insufficiency OR CRF OR end stage kidney disease OR ESKD OR end stage renal disease OR ESRD OR dialysis) AND (pentoxifylline OR oxipentifylline OR trental)) |
