# Supplementary material for: Pentoxifylline for Anemia in Chronic Kidney Disease: A Systematic Review and Meta-Analysis
Source: PLoS One. 2015 Aug 3;10(8):e0134104. doi: 10.1371/journal.pone.0134104 (PMC4523191; doi:10.1371/journal.pone.0134104)
Supplement: S2 Table — (DOCX) [file pone.0134104.s003.docx]

**Supplementary Table S2.** Risk of bias in randomized controlled trials

| **Study, year (ref)** | **Random sequence generation** | **Allocation concealment** | **Blinding of participants and personnel** | **Blinding of outcome assessors** | **Incomplete outcome data** | **Selective reporting** | **Other sources of bias** |
| --- | --- | --- | --- | --- | --- | --- | --- |
| Navarro et al. 1999 [^18^](#_ENREF_18) | **Low risk** (“randomization performed using a randomization table generated by a computer program”) | **Unclear**  (not stated) | **Unclear**  (not stated) | **Unclear**  (not stated) | **Low risk**  (no drop-out) | **Unclear**  (not stated) | **None known** |
| Perkins et al. 2009 [^19^](#_ENREF_19) | **Low risk** (“randomization performed using a randomization table generated by a computer program”) | **Unclear**  (not stated) | **Low Risk**  (double blind) | **Unclear**  (not stated) | **Low risk**  (Overall drop-out 17.5%; 23% vs. 6% drop-out in PTX vs. controls. Analyses made on a *per protocol* basis) | **Low risk**  (all the specified outcomes have been reported) | **None known** |
| Gonzalez-Espinoza et al. 2012 [^20^](#_ENREF_20) | **Low risk**  (“randomization was performed using a randomization list generated by a computer program”) | **Low risk** (“Controls received one identical starch tablet on the same schedule”) | **Low risk**  (triple blind) | **Low risk**  (triple blind) | **Low risk**  (overall drop-out rate 5%) | **Low risk**  (all the specified outcomes have been reported) | **None known** |
| Mortazavi et al. 2012 [^21^](#_ENREF_21) | **Unclear**  (not stated) | **Unclear**  (not stated) | **Unclear**  (not stated) | **Unclear**  (not stated) | **Unclear**  (drop-out rate not reported) | **Unclear**  (not stated) | **None known** |
| AIONID 2013 [^22^](#_ENREF_22) | **Low risk**  (randomization made by using a permuted block approach) | **Low risk**  (containers were packaged in unidentifiable identical formats with unique ID) | **Low risk**  (double blind) | **Unclear**  (not stated) | **High risk**  (overall drop-out 21%) | **Low risk**  (all the specified outcomes have been reported) | **High risk of funding bias**  (“DaVita and Abbott Nutrition provided  the clinical data and resources for conducting the project”) |
| Antunes et al. 2014 [^23^](#_ENREF_23) | **Unclear**  (not stated) | **Unclear**  (not stated) | **High risk**  (open label) | **High risk**  (open label) | **Unclear**  (drop-out rate not reported) | **Unclear**  (not stated) | **None known** |
| HERO 2015 [^24^](#_ENREF_24) | **Low risk** (“participants were randomly assigned in a 1:1 ratio by an adaptive allocation algorithm”) | **Low risk** (“participants in the experimental arm received pentoxifylline  … whereas those in the control arm received identical matching placebo”) | **Low risk**  (triple blind) | **Low risk**  (triple blind) | **High risk**  (overall drop-out rate 16.9% but drop out were 23% vs. 11 % in PTX vs. control; data analyzed on a ITT basis) | **Low risk**  (all the pre-specified outcomes have been reported) | **Low risk of funding bias**  (The trial was funded by industries which had no role in study design, analysis, and interpretation of data) |

**Legend:** ITT: intention to treat; PTX: pentoxifylline
